# Commentary on Postoperative penile prosthesis pain: is it worse in diabetic patients?

**DOI:** 10.1038/s41443-020-0252-6

**Published:** 2020-03-18

**Authors:** John T. Sigalos, Sriram V. Eleswarapu, Jesse N. Mills

**Affiliations:** Department of Urology, David Geffen School of Medicine, University of California, Los Angeles, Los Angeles, CA USA

**Keywords:** Risk factors, Quality of life

The United States opioid crisis has become a major public health and media concern over the last few years. Although poor personal decision-making accounts for a large part of the crisis, physician prescribing also plays a significant role in addiction. Even less invasive cases such as transurethral resection of the prostate can lead to chronic unprescribed opioid use (~5%) [1]. Guidelines on appropriate postoperative pain management from the American Urological Association for the most common urologic procedures do not exist. This is likely due to a lack of robust data to determine what is the ideal compromise between opioid stewardship and effective postoperative pain management. Pain is difficult to study as it is subjective, and the experience of pain varies between patients undergoing the same operation. In addition, pain tolerance and expectations play a large role in patient’s experience of pain in the postoperative period.

Addressing this knowledge gap, Lael Reinstatler et al. in this issue [2] seek to identify the differences in pain experienced by diabetics versus nondiabetics undergoing inflatable penile prosthesis (IPP) placement. There is a dearth of data on postoperative pain management in patients undergoing IPP. Most of the literature in this area has arisen from work describing the effect of perioperative local dorsal penile nerve blocks on postoperative pain management [3, 4]. These studies have shown that local penile blocks, especially those with longer acting formulations, substantially mitigate postoperative narcotic usage and should be used in the placement of IPPs if available. In addition, multi-modal approaches to perioperative analgesia may also reduce opioid use [5, 6].

Lael Reinstatler et al. in this issue highlight the challenges in studying pain management. The surrogate end point for increased pain used in this study was unplanned emergency department (ED) or clinic visits within 30 days with a chief complaint of postoperative pain. While this is an interesting endpoint that can be objectively measured, it is difficult to quantify “how much” pain someone must experience before deciding to seek unplanned care. This decision-making is most likely individualized and highly variable. Potentially, other factors such as time of day of pain onset and amount of pain also likely contribute to the decision to pursue either ED or clinic visit for management of pain. Furthermore, ED versus clinic visits carry a much different cost and burden on the health care system at large. Other objective metrics that would add to our knowledge base and better contextualize this paper include: median number of narcotic tablets taken within 30 days of IPP placement for the cohort at large and for those men with poorly controlled hemoglobin A1c levels; preoperative pain assessments to determine whether diabetic patients started with higher baseline pain on a daily basis; and preoperative assessments of depression and mood disorders, as these are known risk factors for chronic opioid use.

In summary, in order to make more informed decisions about postoperative opioid prescribing, we need to collect more data to create standards of practice for opioid prescribing, and we need to better understand the individualized patient factors such as diabetes, depression, baseline pain, smoking status, and expectations that may alter the patient’s subjective experience and may force the prescriber off the standard path and into more individualized pain management.
